# Trial protocol for the study of recommendation system DiaCompanion with personalized dietary recommendations for women with gestational diabetes mellitus (DiaCompanion I)

**DOI:** 10.3389/fendo.2023.1168688

**Published:** 2023-06-07

**Authors:** Polina Popova, Anna Anopova, Elena Vasukova, Artem Isakov, Angelina Eriskovskaya, Andrey Degilevich, Evgenii Pustozerov, Alexandra Tkachuk, Kristina Pashkova, Natalia Krasnova, Maria Kokina, Irina Nemykina, Tatiana Pervunina, Olga Li, Elena Grineva, Evgeny Shlyakhto

**Affiliations:** ^1^ World-Class Research Center for Personalized Medicine, Almazov National Medical Research Centre, Saint Petersburg, Russia; ^2^ Institute of Endocrinology, Almazov National Medical Research Centre, Saint Petersburg, Russia; ^3^ Institute of Perinatology and Pediatrics, Almazov National Medical Research Center, Saint Petersburg, Russia

**Keywords:** gestational diabetes mellitus, postprandial glycaemic response prediction, personalized nutrition, mHealth, trial protocol

## Abstract

**Background:**

Gestational diabetes mellitus (GDM) is a common complication of pregnancy associated with serious adverse outcomes for mothers and their offspring. Achieving glycaemic targets is the mainstream in the treatment of GDM in order to improve pregnancy outcomes. As GDM is usually diagnosed in the third trimester of pregnancy, the time frame for the intervention is very narrow. Women need to get new knowledge and change their diet very quickly. Usually, these patients require additional frequent visits to healthcare professionals. Recommender systems based on artificial intelligence could partially substitute healthcare professionals in the process of educating and controlling women with GDM, thus reducing the burden on the women and healthcare systems. We have developed a mobile-based personalized recommendation system DiaCompanion I with data-driven real time personal recommendations focused primarily on postprandial glycaemic response prediction. The study aims to clarify the effect of using DiaCompanion I on glycaemic levels and pregnancy outcomes in women with GDM.

**Methods:**

Women with GDM are randomized to 2 treatment groups: utilizing and not utilizing DiaCompanion I. The app provides women in the intervention group the resulting data-driven prognosis of 1-hour postprandial glucose level every time they input their meal data. Based on the predicted glucose level, they can adjust their current meal so that the predicted glucose level falls within the recommended range below 7 mmol/L. The app also provides reminders and recommendations on diet and lifestyle to the participants of the intervention group. All the participants are required to perform 6 blood glucose measurements a day. Capillary glucose values are retrieved from the glucose meter and if not available, from the woman’s diary. Additionally, data on glycaemic levels during the study and consumption of major macro- and micronutrients will be collected using the mobile app with electronic report forms in the intervention group. Women from the control group receive standard care without the mobile app. All participants are prescribed with insulin therapy if needed and modifications in their lifestyle. A total of 216 women will be recruited. The primary outcome is the percentage of postprandial capillary glucose values above target (>7.0 mmol/L). Secondary outcomes include the percentage of patients requiring insulin therapy during pregnancy, maternal and neonatal outcomes, glycaemic control using glycated hemoglobin (HbA1c), continuous glucose monitoring data and other blood glucose metrics, the number of patient visits to endocrinologists and acceptance/satisfaction of the two strategies assessed using a questionnaire.

**Discussion:**

We believe that the approach including DiaCompanion I will be more effective in patients with GDM for improving glycaemic levels and pregnancy outcomes. We also expect that the use of the app will help reduce the number of clinic visits.

**Trial registration number:**

ClinicalTrials.gov, Identifier NCT05179798.

## 1 Background

Gestational diabetes mellitus (GDM) is a common endocrine disorder complicating up to 9-26% of pregnancies, if diagnosed according to IADPSG consensus panel-recommended criteria (1). GDM is associated with adverse pregnancy outcomes, including but not limited to increased rate of cesarean section, macrosomic infants, shoulder dystocia and birth trauma (2). In long term perspective, GDM is associated with an increased risk of obesity, type 2 diabetes mellitus and cardiovascular diseases in mothers and their offspring (3).

Maintaining normal glycaemic levels in pregnancy is essential to prevent adverse pregnancy outcomes and to break the vicious cycle of intergenerational transmission of predisposition to metabolic diseases (4, 5).

Diet is the mainstay of the treatment of GDM. As GDM is usually diagnosed in the third trimester of pregnancy, the time frame for the intervention is very narrow. Women need to get new knowledge and to change their diet very quickly. Thus, the development of effective methods of selecting optimal meal composition to prevent elevated postprandial glycaemic response (PPGR) is important for the treatment of patients with GDM.

Usually, patients with GDM require additional frequent visits to healthcare professionals. Recommender systems based on artificial intelligence could partially substitute healthcare professionals in the process of educating and controlling women with GDM, thus reducing the burden for the women and healthcare systems.

We have developed a mobile-based personalized recommendation system DiaCompanion I with data-driven real time personal recommendations focused primarily on PPGR prediction. The process of developing algorithms for predicting parameters of PPGR [1-hour postprandial blood glucose level (BG60), incremental area under the glycaemic curve 2 hours after meal (iAUC120)] using regression models constructed from anthropometry and laboratory examination data, medical history, questionnaires and food diaries of pregnant women with GDM is described elsewhere as well as the prototype of the recommendation system DiaCompanion (6–8).

Using the developed models, adequate accuracy in predicting PPGRs for women with GDM was obtained (7). The model for predicting peak blood glucose levels after meals, with a mean absolute error of 0.48 mmol/l, formed the basis of the recommendation block of the DiaCompanion I mobile application developed by the authors.

The interactive mobile app, DiaCompanion I, provides real-time prediction of GDM and recommends changes in food intake to prevent hyperglycaemia. The mobile app includes a recommendation block based on recommendations and guidelines for the management of patients with GDM.

A model built into the app predicts what blood glucose levels a woman with GDM will have after a particular meal and, if high blood glucose levels are predicted, estimates the reasons for the increase and shows dietary recommendations to prevent hyperglycaemia.

Currently there is growing interest in digital health solutions for the management of women with GDM. A recent meta-analysis has shown benefits of using m-health solutions to improve glycaemic control and pregnancy outcomes in GDM (9). However, comparison is complicated by the differences among the developed technologies and by little agreement on the key components of interventions. Only a few digital health applications have been integrated in clinical practice (10). To the best of our knowledge, no one of the published studies used PPGR prediction in their digital health solutions for women with GDM.

We aim to clarify the effect of using mobile-based personalized recommendation system DiaCompanion I on glycaemic levels and pregnancy outcomes in women with GDM.

## 2 Methods and analysis

### 2.1 Study design and setting

**Figure 1:**
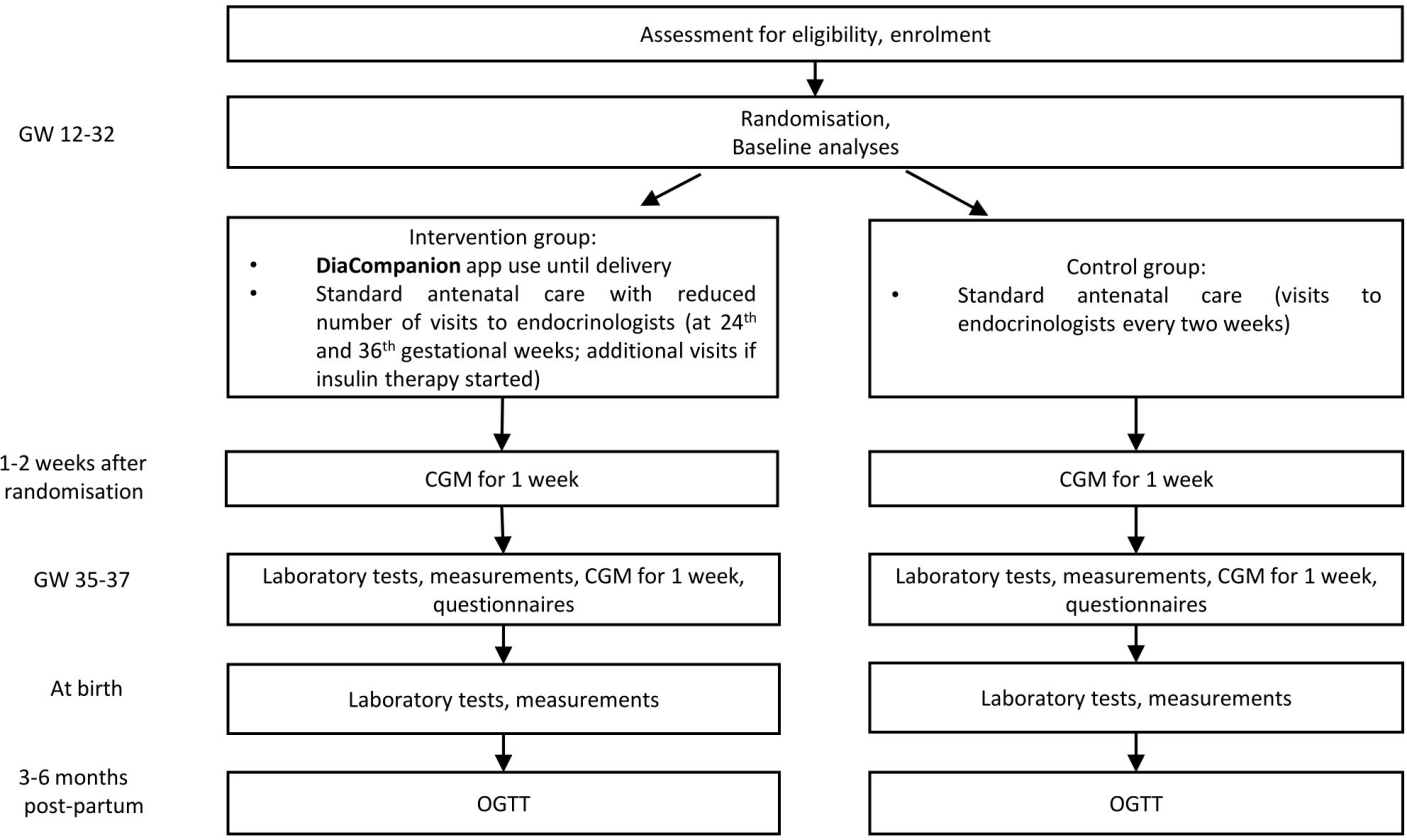
Design of the study.

This study is an interventional open-labeled randomized controlled trial. A total of 216 women with GDM will take part in this trial performed in the Almazov National Medical Research Centre. Patients are randomized 1:1 to the intervention or control group. The intervention group receives DiaCompanion I app and standard antenatal care with the reduced number of visits to endocrinologists. The control group receives standard antenatal care. Patients participate in the study from recruitment (between 12 and 32 completed weeks of pregnancy) until delivery and will be invited to the follow-up visit at 3-6 months postpartum. The study protocol follows the Standard Protocol Items: Recommendations for Interventional Trials (SPIRIT) (11). Study design and data collection are shown in **Figure 1**. Please find the detailed information on the intervention and data collection in the sections below.

### 2.2 Participants

Pregnant women with GDM are invited to participate if they are not more than 4 weeks after confirmation of GDM diagnosis (after a clinical visit when patient is informed of the diagnosis and prescribed with diet). GDM is diagnosed according to IADPSG (International Association of the Diabetes and Pregnancy Study Groups) criteria, i.e. fasting plasma glucose between 5.1 mmol/L and 6.9 mmol/L after the 6th week of gestation and/or 1-hour plasma glucose value after 75 g oral glucose tolerance test (OGTT) ≥ 10.0 mmol/L and/or 2-hour plasma glucose value between 8.5 mmol/L and 11.0 mmol/L at the 24-31^st^ week of gestation. According to Russian national guidelines, OGTT is performed between 24^th^-28^th^ week of gestation and in exceptional cases can be performed up to the 32^nd^ gestational week. The inclusion and exclusion criteria are listed in **Table 1**.

**Table 1:** Inclusion and exclusion criteria.

Inclusion Criteria	Exclusion Criteria
• Women diagnosed with GDM according to IADPSG • Age >18 years • Gestational age >= 12 and < 32 weeks • No more than 4 weeks after confirmation of GDM diagnosis • Singleton pregnancy • The ability to navigate an app • Provided informed consent	• Preexisting diabetes of any type before the current pregnancy • Need for insulin therapy at the time of screening • Heart failure • Chronic kidney disease • History of bariatric surgery • Use of long-term systemic corticosteroids • Impaired mobility • Known fetal malformations • Concomitant participation in other clinical trials

### 2.3 Recruitment

Participants are recruited from the outpatient department of the Perinatal Center of the Almazov National Medical Research Center and from antenatal clinics of Saint Petersburg. Local obstetricians and endocrinologists inform about the possibility to participate in the DiaCompanion I trial and share flyers with pregnant women during their first visit after GDM confirmation. Pregnant women with GDM willing to participate are screened by the team members (endocrinologists of the Almazov Centre) for eligibility. Those women that meet the inclusion/exclusion criteria listed in **Table 1** and sign the consent form are included into the study. The consent form is signed by the woman and a qualified health-care professional with delegated authority in two copies, one for the woman and one for the study records. We started the recruitment in January 2022 and we plan to continue until November 2024.

### 2.4 Randomization

Women are randomized individually in an equal ratio of standard care (control) to DiaCompanion I app use (intervention). Randomization is performed with a randomized computer-generated number table generated by the statistician using the randomizeR package in R (12) prior to the first participant’s baseline visit. The randomization list is available only to research officers. А research member in the clinic will call research officers to obtain group allocation during the baseline visit after obtaining the consent form.

### 2.5 DiaCompanion I description

Intervention group will use the mobile app DiaCompanion I with personalized dietary recommendations. DiaCompanion I was developed in 2021. In the previous study, we developed and tested in clinical practice the mobile application DiaCompanion for data collection and exchange (8, 13, 14). It allows tracking of food intakes, physical activities, insulin doses, urine ketones and blood glucose measurements performed by the participants. The data accumulated during the study on the postprandial glycaemic response (PPGR) of GDM patients to certain meals in various situations allowed us to develop predictive models for assessing the risk of a high PPGR after a meal based on data on the current meal, meals during the last day, and individual characteristics of the patient (7).

**Figure 2:**
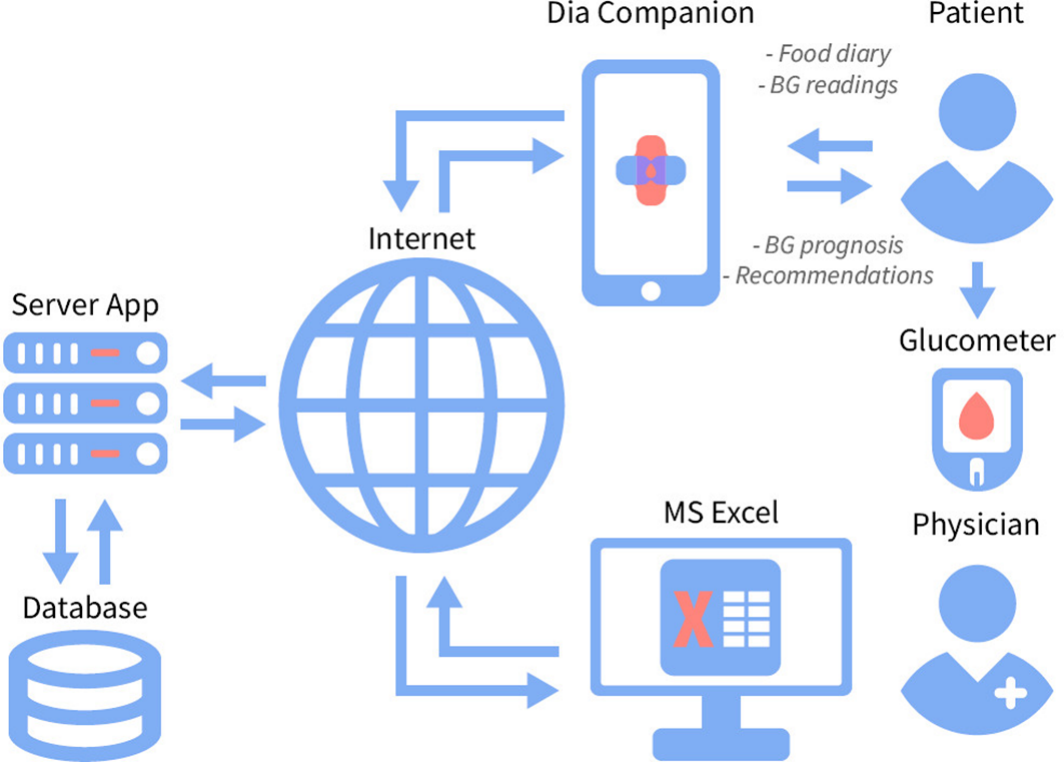
The recommendation system DiaCompanion I with personalized dietary recommendations for women with GDM.

In addition to the functions of DiaCompanion, the DiaCompanion I app contains a recommendation block which predicts the PPGR based on the personal parameters, current planned meal content and information from the diary for the last few days (**Figure 2**). Then, depending on the predicted postprandial BG level, the application generates one of the pop-up messages with recommendations. In the case of predicting a BG above target (> 7,0 mmol/L), pop-up messages contain recommendations on how to prevent high postprandial glucose after the planned meal. The messages include recommendations to exclude or reduce the number of foods with a high glycaemic index (GI), reduce the number of carbohydrates in the index meal or during snacks, and make changes to the meal. As participants receive predicted BG level and corresponding recommendations at the time of planning a food intake, they can change the content of the planned meal in the app till the predicted BG value falls within the target range. In the case of predicting a PPGR within the normal range, a message appears about a successfully added record. The app also analyzes potential reasons for a high predicted BG level. If the reason is beyond the high glycaemic load of the planned meal, recommendations on additional physical activity, reduction of carbohydrates in snacks, increasing the consumption of fibers and/or seeking advice from endocrinologist may appear.

The application implements a system for visualizing the quantitative effect on the PPGR of individual products and dishes, taking into account their GI: green - dishes with a GI of less than 20, yellow - from 20 to 69, red - 70 and higher. Usually, a GI below 55 is considered to be «low» (15). However, foods with a GI slightly below 55 can also induce a high PPGR if consumed in substantial amounts. For the purpose of our study, we marked by green color the foods which are likely not to increase the level of glycemia in any amounts consumed.

The application contains teaching materials including general information about GDM, nutrition principles for patients with GDM, information about the GI of foods, dietary fibers and their importance in the diet, and recommendations on physical activity.

The application also contains an alert system in the form of pop-up messages on a woman’s device with a reminder of the insufficient number of diary records entered for the previous days, a reminder to send a diary to the doctor, and generates reports for the doctor.

DiaCompanion I is represented by two mobile versions developed for Android and iOS devices, as well as a cross-platform web version. The implementation features of the Android and iOS applications also allow them to be used on computers with preset Java Runtime Machine. The experience gained in developing the web version influenced the choice of tools for creating a server to exchange information between different user devices within the same account.

**Figure 3:**
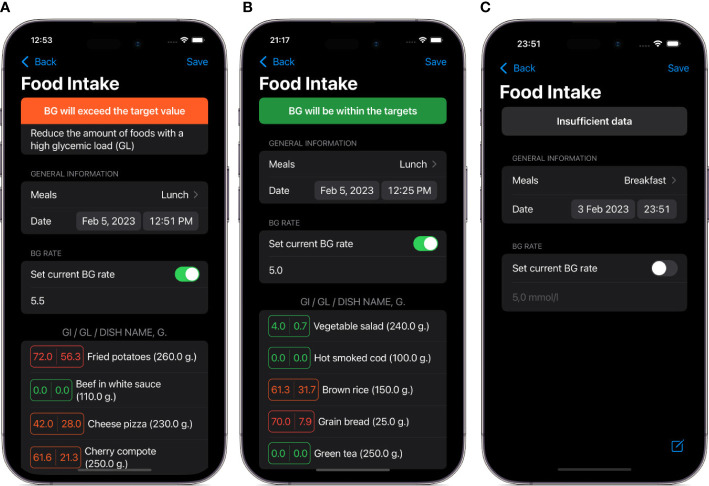
Screenshots of main views in the DiaCompanion I app. **(A)** a view of food intake data before entering planned food items and current blood glucose level. **(B)** an example of a meal with a high level of predicted postprandial blood glucose. **(C)** an example of a meal with a level of predicted postprandial blood glucose within targets.

The app contains plenty of screens with a wide range of functions, including the main menu, user input forms, record management and information, user settings, data export form, and help. Some of these screens are presented in **Figure 3**.

The built-in food database, collected from open sources (including the Scientific Research Institute of Nutrition of the Russian Academy of Medical Sciences and the USDA food databases) enables tracking 27 food parameters (macronutrients and micronutrients). Additionally, glycaemic indexes were assigned to all food items in the food database using a standardized protocol (6).

Patients can add their own dishes to the app by composing them from the ingredients presented in the built-in database. The automatic algorithm calculates their nutrient profile.

**Figure 4:**
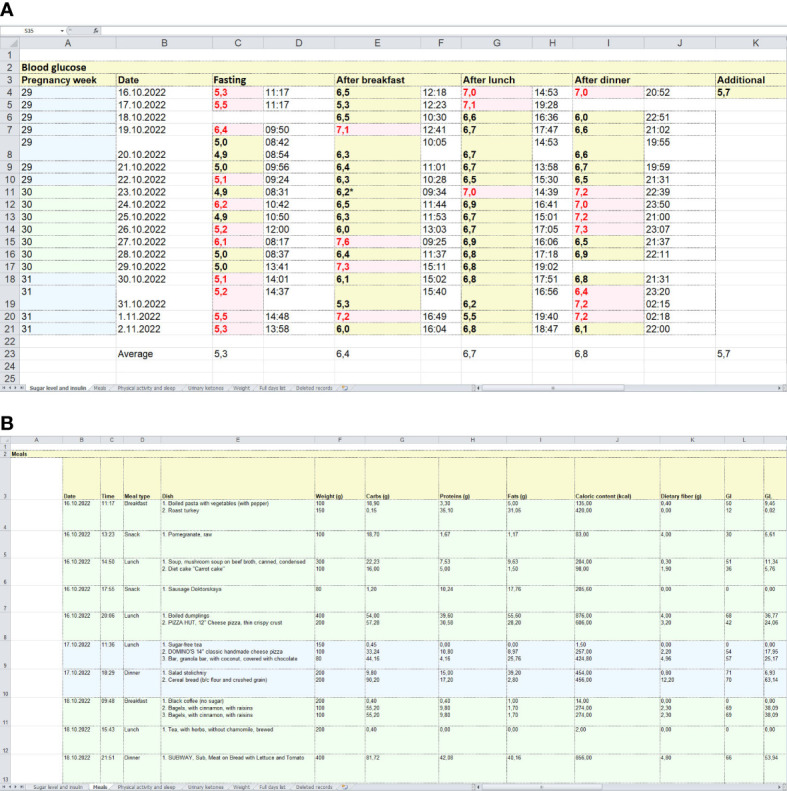
Examples of standardized reports exported from the app. **(A)** = a spreadsheet with glucose levels measured by a glucometer. **(B)** = an example of a spreadsheet with food intake records, including food items (dishes), their weights, macronutrient contents, GI and glycaemic loads.

The app allows the users to export data in Excel spreadsheets and store them on their devices as well as send them remotely to physicians. An example of a standardized report exported from the app is shown in **Figure 4**.

To synchronize data between patient devices, implement authorization and automatically save user data, a web application for the server based on the Django framework was developed.

Patient data in this system is stored on the servers of the Almazov National Medical Research Centre and is automatically updated every week by downloading data from the patient’s devices. This approach protects patient data by TLS encryption and by avoiding weakly protected communication channels like email. Moreover, the system protects data from unintentional deletion of record history.

### 2.6 DiaCompanion I intervention

Participants in the intervention group are required to enter all food intakes into the app. They are also required to enter blood glucose measurements before main food intakes (breakfast, dinner and lunch) to enable the prediction of postprandial BG level. In case of a high predicted BG they are encouraged to change the amount and/or content of the planned meal in a manner that will lead to a predicted BG within target range.

Women in the intervention group are recommended to send their electronic diaries (standardized reports) to their endocrinologists every week in order to get feedback. The doctors check the food records and glucose levels and send back recommendations on dietary corrections if necessary. In case of revealing indications for insulin therapy, the women are appointed to an office visit within a few days.

The participants of the intervention group have only two scheduled visits to endocrinologists during pregnancy (at baseline and on the 35-37^th^ gestational week (GW)) if they are included after the 20^th^ GW. Those included before the 20^th^ GW are also invited at the 24^th^ GW. Additional visits are appointed in case of the appearance of indications to insulin treatment (repeated fasting capillary glucose of ≥5.1mmol/L or a 1-hour postprandial value of ≥7.0 mmol/L during 1-2 weeks). Two or more abnormal values during 1-2 weeks of monitoring are required to commence insulin treatment according to Russian national guidelines (16).

### 2.7 Standard care

Women in the control group will have a standard of care without a mobile app. According to Russian national guidelines, women diagnosed with GDM receive guidance on diet, physical activity and self-monitoring of BG with capillary glucose meters. They are required to self-record their fasting, pre-prandial and 1-hour postprandial BG values and they are also advised to self-record their food intakes in a paper diary at home. They are attending the maternity clinic every 2-3 weeks where an endocrinologist reviews their readings and diary. In case of repeated fasting capillary glucose of ≥5.1mmol/L or a 1-hour postprandial value of ≥7.0 mmol/L during 1-2 weeks or one reading above 9.0 mmol/L at any time of day, participants are instructed to call the clinic and schedule an additional visit to reconsider indications for insulin treatment.

### 2.8 Proposed outcome measurement

Primary Outcome is the percentage of postprandial capillary glucose values above target (>7.0 mmol/L) in the time frame from randomization up to delivery. The women will be asked to perform 6 measures a day. Capillary glucose values will be retrieved from the glucose meter, and if not available, from the woman’s diary.

Secondary outcome measures are:
1. Rate of patients requiring insulin therapy during pregnancy
⚬ Rate of patients requiring insulin therapy (either basal or prandial)
⚬ Rate of patients requiring prandial insulin therapy
2. Capillary glucose values in the following time frames: within 2 weeks after randomization , at gestational weeks 35-37, and in the period from randomization up to delivery.
⚬ Capillary fasting glucose values, mmol/L
⚬ Capillary preprandial glucose values, mmol/L
⚬ Capillary postprandial glucose values, mmol/L
3. The proportion of women with glucose values within target in the following time frames: within 2 weeks after randomization, at gestational weeks 35-37, and in the period from randomization up to delivery.
⚬ The proportion of women with fasting glucose values within the target
⚬ The proportion of women with postprandial glucose values within the target
4. Mean incremental area under the blood glucose curve 2 hours after meals (iAUC120) according to 7-day continuous glucose monitoring 1-2 weeks after randomization and at the 35-37^th^ GW.
5. Hypoglycaemia
⚬ Severe hypoglycaemia: requiring the assistance of another person to actively correct the level of glycaemia and neurological symptoms
⚬ Documented symptomatic hypoglycaemia: event during which typical symptoms of hypoglycaemia are accompanied by a measured capillary glucose concentration <3.9 mmol/L
⚬ Asymptomatic hypoglycaemia: event not accompanied by typical symptoms of hypoglycaemia but with a measured capillary glucose concentration <3.3 mmol/L
6. Maternal metabolic parameters at 35-37^th^ GW (the level of HbA1c, Fasting insulin, Fasting glucose, HOMA-IR index, serum triglyceride levels).
7. Gestational weight change by the time of delivery
⚬ Gestational weight change during pregnancy, kg
⚬ Gestational weight change between inclusion and delivery, kg
8. Large (>90th percentile) for gestational age infant
9. Small for gestational age (< 10th percentile) infant
10. Birth weight, kg
11. Birth weight ≥ 4000 g; ≥ 4500 g (yes/no)
12. Neonatal hypoglycaemia defined as glucose levels <2,2 mmol/L in the first 4 hours of life or <2,5 mmol/L at 4–24 hours of age (yes/no) (17).
13. Shoulder dystocia (yes/no)
14. Birth injury, any of the following: plexus injury, clavicle, humeral, or skull fracture (yes/no)
15. Preterm delivery (yes/no)
16. Apgar score at 1 and 5 minutes from birth
17. Low Apgar score: 5-min Apgar score < 7 (yes/no)
18. Jaundice requiring phototherapy (yes/no)
19. Neonatal respiratory distress syndrome (yes/no)
20. Admission to neonatal intensive care unit during the three days following birth (yes/no)
21. Umbilical cord blood C-peptide, ng/mL
22. Cesarean delivery rate
23. Induction of labor rate
24. Need for operative vaginal delivery rate (forceps or vacuum-assisted vaginal delivery)
25. Preeclampsia (blood pressure ≥ 140/90 mmHg on two measurements four hours apart and proteinuria of at least 300 mg/24 hours or 3+ or more on dipstick testing or proteinuria/creatinuria >30 in a random urine sample)
26. Pregnancy-induced hypertension in women with no known hypertension before pregnancy (blood pressure ≥ 140/90 mmHg on two measurements four hours apart without proteinuria, and required anti-hypertensive therapy)
27. The number of in-patient visits to endocrinologists in the time frame from randomization to delivery.
28. Results of oral glucose tolerance test 3 months postpartum.
29. Acceptance/satisfaction of 2 strategies at 35-36^th^ GW (Evaluation of the patient satisfaction with their treatment for GDM with a scale: give a score of 0 to 100: 0 not satisfied; 100 totally satisfied).
30. Patient satisfaction evaluated through a questionnaire at 35-36^th^ GW.

We will use a 10-question questionnaire with multiple-choice questions, questions with numeric answers on a scale from 0 (fully unsatisfied) to 10 (fully satisfied), and open-ended questions.

The timetable of the planned visits and measurements is shown in **Table 2**.

**Table 2:** Measurements timeline.

Measurements for both groups	Baseline	24^th^ GW*	35-36^th^ GW	Birth		Postpartum (3- 6 months)
Questionnaires (SF36, STAI, treatment attitude)	**x**		**x**			
Patient satisfaction questionnaire			**x**			
Physical examination (height, weight, blood pressure, oedema)	**x**	**x**	**x**			**X**
Medtronic CGM sensors	**x**		**x**			
Laboratory blood tests	**x**		**x**			
Cord blood samples and HUVECs				**x**		
Infant’s anthropometry, Apgar score, BG 1 hour after birth				**x**		
OGTT						**x**
Fecal, urine and saliva samples	**x**		**x**			
BG readings (glucometer)	**x**	**x**	**x**			
Measurements only for the intervention group:						
Data from DiaCompanion I app (BG readings, food tracker)	**x**	**x**	**x**			
Technology acceptance questionnaire			**x**			

*Visit at the 24^th^ GW will be scheduled for the participants included before the 20^th^ GW.

GW, gestational week; SF36, Short Form-36, STAI, State-Trait Anxiety Inventory, CGM, continuous glucose monitoring; BG, blood glucose; HUVECs, human umbilical vein endothelial cells; OGTT, oral glucose tolerance test.

### 2.9 Data analysis

The analyses will be performed on the intention-to-treat (ITT) basis, including all randomized patients. We will also perform a per-protocol analysis excluding women who did not use the DiaCompanion I app for at least 50% of expected meals to explore the efficacy of the system under optimal use.

Quantitative data will be assessed for normality using the Kolmogorov-Smirnov test. Log transformation could be used where appropriate. The data will be presented as mean and SD for normally distributed parameters and as median with quartiles for non-normally distributed characteristics. The distribution of binary or ordinary characteristics will be described as N (%).

The significance of differences in scale parameters (such as the percentage of postprandial capillary glucose values above target, the number of in-patient visits, etc.) between the control and intervention groups will be assessed by the Student’s t-test for normally distributed ones and Mann-Whitney test for ordinal and non-normally distributed characteristics. To compare the distribution of binary or ordinary characteristics (ex., rate of patients requiring insulin therapy during pregnancy, LGA, SGA, etc.) the χ2 criterion or Fisher’s exact test will be used. The Spearman correlation coefficient will be used to assess the relationship between two variables. We will use ANOVA repeated or nonparametric analogue for the analysis of repeated measurements (gestational weight change). The critical level of significance of the null statistical hypothesis will be assumed to be 0.05.

### 2.10 Sample size

The sample size for the RCT was calculated as the number of participants needed to test the statistical significance of the difference between the two means (reduction in mean percent of BG readings exceeding target glycaemic levels from 15 to 11%, SD 10%) at a significance level of 0.05 and with a power of 0.80 for d/SD values = 0.4, including 25% dropout.

## 3 Discussion

Our mobile-based recommendation system DiaCompanion I differentiates from existing GDM apps by the ability to predict postprandial BG level at the time of food intake planning and by providing real-time personalized recommendations on the ways of hyperglycemia prevention.

There are published and ongoing trials of the effect of personalized diets by prediction of glycaemic responses on glycaemic control in prediabetes and type 2 diabetes mellitus. However, these analogues are not intended for pregnant women (18, 19).

The authors of a recent review investigating the awareness and use of the smartphone applications (app) with respect to management of GDM concluded that mobile apps provided personalized health care services, patient care improvement, and enhanced patient’s compliance toward blood glucose monitoring and treatment (20). A relatively large RCT by Mackillop et al. (21) reported less preterm births and fewer cesarean deliveries despite the fact that there was no significant change in BG levels between the control and the intervention groups.

In another scoping review, the authors investigated mHealth apps intended for use with GDM and concluded that there were limited mHealth apps for GDM that incorporated AI or AI-based decision support (22).

It is well known that learning how to manage the results of self- tracking and adhering to a diet requires efforts from women with GDM, as associations between lifestyle, nutrition and PPGR are complex and difficult to identify, especially during the first weeks after GDM diagnosis (23). As DiaCompanion I provides recommendations at each time when a high BG is predicted, we expect the most significant difference in glucose control parameters within the first weeks after GDM diagnosis.

Our system does not consist of an app only, as participants in the intervention group are supposed to generate diary reports from the app weekly and send it to their physicians. Using this approach, we substitute most of in-person communications with a feedback based on diary analysis by means of return messages. We believe that the combination of automated feedback from the app before each meal intake with weekly feedback from a healthcare professional will further increase the efficacy of the intervention while reducing the number of in-patients visits. Apart from reducing the burden on health care facilities, we expect a reduction in total time spent for patients communicating by endocrinologists or other health care providers dealing with BG and diet control in patients with GDM.

However, this approach also poses challenges. It is difficult to differentiate the influence from automated recommendations based on PPGR prediction and the influence of weekly medical feedback. Another limitation of our study is the lack of BG prediction and insulin doses adjustment for patients treated with insulin. Women requiring insulin will be recorded and followed until delivery but they will not be able to use the prediction function. It is supposed to work incorrectly in such patients because we have not included insulin treated women in the population used for the development of PPGR prediction models. It is a task for our future research to develop such models.

## Trial status

The study protocol was first posted on January 5, 2022, and enrollment has started on January 12, 2022. The estimated primary completion date is January 2025.

## Ethics statement

The studies involving human participants were reviewed and approved by Ethics committee of the Almazov National Medical Research Centre. The patients/participants provided their written informed consent to participate in this study.

## Author contributions

Conceptualization: PP, ES. Methodology: PP, EP, TP and EG. Investigation: AT, EV, AA, KP, NK, IN, MK and OL. Resources: TP. Data curation: AT, AA and EV. Writing—original draft preparation: PP and AE. Writing—review and editing: PP, EG, and TP. Statistical analysis: PP, AI, AD, AE. Supervision: EG and ES. Project administration: PP. Funding acquisition: ES. All authors contributed to the article and approved the submitted version.
